# Antibiotic Use In Utero and Early Life and Risk of Chronic Childhood Conditions in New Zealand: Protocol for a Data Linkage Retrospective Cohort Study

**DOI:** 10.2196/66184

**Published:** 2025-02-28

**Authors:** Sharan Ram, Marine Corbin, Andrea 't Mannetje, Amanda Eng, Amanda Kvalsvig, Michael G Baker, Jeroen Douwes

**Affiliations:** 1 Centre for Public Health Research Massey University Wellington New Zealand; 2 Department of Public Health University of Otago Wellington New Zealand

**Keywords:** early childhood, chronic childhood conditions, antibiotics, data linkage, study protocol, routine data

## Abstract

**Background:**

The incidence of many common chronic childhood conditions has increased globally in the past few decades, which has been suggested to be potentially attributed to antibiotic overuse leading to dysbiosis in the gut microbiome.

**Objective:**

This linkage study will assess the role of antibiotic use in utero and in early life in the development of type 1 diabetes (T1D), attention-deficit/hyperactive disorder (ADHD), and inflammatory bowel disease.

**Methods:**

The study design involves several retrospective cohort studies using linked administrative health and social data from Statistics New Zealand’s Integrated Data Infrastructure. It uses data from all children who were born in New Zealand between October 2005 and December 2010 (N=334,204) and their mothers. Children’s antibiotic use is identified for 4 time periods (at pregnancy, at ≤1 year, at ≤2 years, and at ≤5 years), and the development of T1D, ADHD, and inflammatory bowel disease is measured from the end of the antibiotic use periods until death, emigration, or the end of the follow-up period (2021), whichever came first. Children who emigrated or died before the end of the antibiotic use period are excluded. Cox proportional hazards regression models are used while adjusting for a range of potential confounders.

**Results:**

As of September 2024, data linkage has been completed, involving the integration of antibiotic exposure and outcome variables for 315,789 children. Preliminary analyses show that both prenatal and early life antibiotic consumption is associated with T1D. Full analyses for all 3 outcomes will be completed by the end of 2025.

**Conclusions:**

This series of linked cohort studies using detailed, complete, and systematically collected antibiotic prescription data will provide critical new knowledge regarding the role of antibiotics in the development of common chronic childhood conditions. Thus, this study has the potential to contribute to the development of primary prevention strategies through, for example, targeted changes in antibiotic use.

**International Registered Report Identifier (IRRID):**

DERR1-10.2196/66184

## Introduction

The incidence of many chronic childhood conditions such as type 1 diabetes (T1D), attention-deficit/hyperactive disorder (ADHD), and inflammatory bowel disease (IBD) has increased globally in the last 2 decades [1]. The total global incidence of childhood and adolescent T1D is larger than previously estimated, with nearly 1 in 2 children currently undiagnosed, with most underdiagnoses occurring in low-income countries [2]. Current global incidence estimates range from 128,900 to 149,500 per annum [3,4]. For ADHD, although recent geographical estimates are unavailable, the highest incidence rates are reported in countries with a higher sociodemographic index [5]. The global incidence of IBD is increasing steadily and varies greatly by geographical areas, with the highest annual pediatric incidence of IBD reported to be 23/100,000 person-years in Europe, 15.2/100,000 in North America, and 11.4/100,000 in Asia/the Middle East and Oceania [6].

In New Zealand, the annual incidence of T1D is reported to be 23/100,000, and this is increasing by 4.1% annually [7]. It is estimated that 2%-5% of school-age children in New Zealand are affected by ADHD [8], with a recent study showing that the total ADHD medication dispensing prevalence had almost doubled from 516/100,000 in 2007-2008 to 996/100,000 in 2016-2017, with the highest dispensing prevalence reported for those aged 7-17 years [9]. In the case of IBD, recent New Zealand estimates have shown age-specific incidence rates of 39.5/100,000, which is 1.6-fold greater than what was measured 10 years earlier—this is among the highest in the world [10]. Research indicates significant ethnic differences in T1D and IBD, with European children having higher rates than Māori and Pacific children [7,11]. Furthermore, ADHD prevalence and treatment access vary by socioeconomic status [12].

The etiology of these diseases is not well understood, but environmental factors, genetics, immune-regulatory pathways, and microbial exposures are considered important [13,14]. Early life gut microbiome development is a critical window for immune and neurodevelopment. In early childhood, establishing a healthy microbiome is vital for shaping the immune system and influencing neurodevelopmental outcomes [15]. Microbial colonization begins at birth and evolves with a diverse gut microbiome linked to better health outcomes. Antibiotic use during this period may also be a risk factor that can disrupt the microbiome development by depleting beneficial bacteria, leading to dysbiosis [15,16], which may impair immune training and increase susceptibility to these chronic conditions [17].

Several studies have assessed associations between antibiotic use and the development of these chronic conditions during the prenatal period and early life years [18-20], but results have been inconsistent, with some showing positive associations [20] and others showing no association [21-23]. These inconsistencies may, at least in part, be explained by limitations in study design. For example, antibiotic use is often assessed through recall, which is vulnerable to bias. Moreover, studies often rely on short-term prescription history prior to disease onset, which may result in issues of reverse causality (if antibiotics were prescribed to treat early symptoms of the disease itself) [24,25]. Further, studies have often focused on only one specific class of antibiotics without considering the full spectrum of antibiotics used [25]. Importantly, most research has been conducted in Europe (particularly Scandinavia) and the United States, and it remains unclear whether results can be extrapolated to other parts of the world, including New Zealand, which, compared to other Organisation for Economic Co-operation and Development countries, including Scandinavia [26], is known to have a very high use of antibiotics among children [27].

The series of linked cohort studies for which the methods are described in this protocol paper will assess associations between prenatal and early life antibiotic use and the development of childhood T1D, ADHD, and IBD. These studies using detailed, complete, and systematically collected antibiotic prescription data will provide critical new knowledge regarding the role of antibiotics in the development of these common chronic childhood conditions. Thus, this study has the potential to contribute to the development of primary prevention strategies through, for example, targeted changes in antibiotic use. The central hypothesis of this study is that early life antibiotic use is associated with the development of childhood T1D, ADHD, and IBD.

## Methods

### Study Design, Setting, and Population

The study design involves several retrospective cohort studies using linked administrative health and social data from Statistics New Zealand’s Integrated Data Infrastructure (IDI) [28]. Antibiotic use and T1D, ADHD, and IBD are defined as described below. To date, data for all children born in New Zealand between October 2005 and December 2010 (N=334,204) and their mothers have been extracted from the Department of Internal Affairs birth data in the IDI. Children’s antibiotic use has been defined for 4 time periods (at pregnancy, at ≤1 year, at ≤2 years, and at ≤5 years). The development of T1D, ADHD, and IBD (which consists of Crohn disease and ulcerative colitis) has been measured from the end of the antibiotic use periods until death, emigration, or the end of the study in 2021, whichever came first, accumulating approximately 3,000,000 person-years. Children who emigrated overseas or died before the end of the antibiotic use period have been excluded from the analysis, as they cannot be followed up for the occurrence of these chronic childhood conditions. At the end of follow-up, children had reached the age of 11-16 years.

### Data Sources

The IDI is a database of deidentified administrative and survey data about people and households in New Zealand [28]. It includes data about health, education, income, social support payments, migration, and other life events, which can be linked at the individual level. The IDI provides a longitudinal record of events and is a growing resource. As of September 2018, the IDI holds over 166 billion pieces of information from more than 14 organizations [28,29]. Table 1 lists the datasets that are being used for this study with a brief description of the data and the variables extracted from these datasets.

**Table 1:** Data collections available within the integrated data infrastructure for linking cohorts’ demographic data with antibiotic use and selected health outcomes [30].

Data collections	Descriptions	Characteristics/variables extracted
Births (from 1840): This data collection was used to define the cohort and identify the mothers of the children.	This collection holds all births in New Zealand, including month and year of birth, sex, ethnicity, first and second parent as recorded on birth registration, and their sex, age, ethnicity, type of relationship, weight at birth, gestation, and their age.	Sex, date of birth, birth weight, ethnicity
Pharmaceutical data (from 2005): This data collection was used to analyze antibiotic prescription.	This collection holds claim and payment information from pharmacists for subsidized medicines, including Pharmaceutical Management Agency (PHARMAC^a^) identifier of primary active chemical ingredient, quantity, number of repeats, and date of dispensing.	Date of prescription dispensing, Anatomical Therapeutic Chemical codes for medicines, including antibiotics and treatments for T1D^b^, ADHD^c^, and IBD^d^
Maternity (from 2003): This data collection was used to calculate the gestational period and maternal age at birth and to identify the mode of delivery.	The National Maternity Collection provides statistical, demographic, and clinical information about selected publicly funded maternity services up to 9 months before and 3 months after a birth.	Mothers’ date of birth, ethnicity, last date of menstruation, mode of delivery, maternal age at delivery
Mortality (from 1998): These data were used to identify the date of death.	This collection holds the underlying cause of death for all deaths registered in New Zealand using the International Classification of Diseases, Tenth Revision, Clinical Modification codes, including all registered fetal deaths and date of death.	Date of death
International travel and migration (from 1997): This data collection was used to identify children who emigrated from New Zealand.	This collection holds arrival and departure records and migration records.	Date of departure
Laboratory claims (from 2003): This data collection was used to obtain laboratory testing information.	This collection holds primary-care test subsidies.	Laboratory test(s) conducted (results of tests are not available), including testing for T1D, ADHD, and IBD
NNPAC^e^ (from 2007): This data collection was used to identify any diagnosis procedure for nonadmitted patients.	NNPAC provides national consistent data on nonadmitted patient (outpatient and emergency department) activity.	Diagnosis for various health conditions, including T1D, ADHD, and IBD
Publicly funded hospital discharges (from 1998): This data collection was used to identify the principal and additional reasons for hospitalization and procedure performed during hospital stay.	This collection contains summarized information detailing publicly funded hospital discharges and procedures by New Zealand hospitals using the codes of the International Classification of Diseases, Tenth Revision, Clinical Modification.	Disease/procedure classification, diagnosis for various health conditions, including T1D, ADHD, and IBD.

^a^PHARMAC is a government agency in New Zealand responsible for managing the funding and procurement of pharmaceuticals and medical devices.

^b^T1D: type 1 diabetes.

^c^ADHD: attention-deficit/hyperactivity disorder.

^d^IBD: inflammatory bowel disease.

^e^NNPAC: National Non-Admitted Patient Collection.

### Definition of Antibiotic Exposure

Antibiotic exposures in utero and for the first 5 years of life are identified for all cohort members born between October 2005 to December 2010 from pharmaceutical data. Dispensing dates and dose and number of purchases are identified, and each antibiotic prescription is categorized by (1) class, according to the Anatomical Therapeutic Chemical classification J01 “Antibiotics for systemic use” (eg, penicillins, cephalosporins, sulfonamides); (2) spectrum, that is, broad or narrow; and (3) whether antibiotics target Gram-positive or Gram-negative bacteria or both. These categorizations of individual antibiotics are provided in Table 2 [31].

**Table 2:** Antibiotics by class and spectrum of activity [31].

Class of antibiotics, chemical name of antibiotic used among the cohort			Spectrum of activity (narrow/broad)		Antibiotics targets Gram-positive/Gram-negative bacteria
**Cephalosporins and cephamycins**					
	Cefaclor monohydrate	Moderate		Both	
	Cefalexin	Moderate		Both	
	Cefamandole nafate	Broad		Both	
	Cefazolin	Moderate		Both	
	Cefoxitin sodium	Moderate		Both	
	Ceftazidime	Broad		Both	
	Ceftriaxone	Broad		Both	
	Cefuroxime axetil	Moderate		Both	
	Cefuroxime sodium	Moderate		Both	
	Cephalothin sodium	Broad		Both	
	Cephradine	Broad		Both	
**Macrolides**					
	Azithromycin	Broad		Positive	
	Clarithromycin	Broad		Positive	
	Erythromycin	Broad		Positive	
	Erythromycin (as lactobionate)	Broad		Positive	
	Erythromycin estolate	Broad		Positive	
	Erythromycin ethyl succinate	Narrow		Positive	
	Erythromycin stearate	Narrow		Positive	
	Roxithromycin	Broad		Positive	
**Other antibiotics**					
	Aztreonam	Broad		Negative	
	Chloramphenicol	Broad		Both	
	Chloramphenicol sodium succinate	Broad		Both	
	Ciprofloxacin	Broad		Both	
	Clindamycin	Broad		Both	
	Colistin sulfomethate	Broad		Positive	
	Fleroxacin	Broad		Both	
	Framycetin sulfate	Broad		Both	
	Gentamicin sulfate	Broad		Both	
	Imipenem	Broad		Both	
	Levofloxacin	Broad		Both	
	Lincomycin	Broad		Both	
	Lincomycin hydrochloride	Narrow		Positive	
	Moxifloxacin	Broad		Both	
	Neomycin sulfate	Broad		Both	
	Ofloxacin	Broad		Both	
	Paromomycin	Broad		Positive	
	Pyrimethamine	Broad		Both	
	Sodium fusidate (fusidic acid)	Narrow		Both	
	Spectinomycin hydrochloride	Moderate		Both	
	Spiramycin	Broad		Both	
	Sulfadiazine sodium	Broad		Both	
	Sulfadiazine	Broad		Both	
	Tobramycin	Broad		Both	
	Triacetyloleandomycin	Broad		Positive	
	Trimethoprim	Broad		Both	
	Trimethoprim with sulfamethoxazole (cotrimoxazole)	Broad		Both	
	Vancomycin	Narrow		Positive	
**Penicillins**					
	Amoxicillin	Broad		Both	
	Amoxicillin with clavulanic acid	Broad		Both	
	Amoxicillin clavulanate	Broad		Both	
	Benzathine benzylpenicillin	Narrow		Both	
	Benzylpenicillin sodium (Penicillin G)	Narrow		Both	
	Dicloxacillin	Narrow		Both	
	Flucloxacillin	Narrow		Both	
	Flucloxacillin magnesium	Narrow		Both	
	Penicillin G benzathine (Benzathine benzylpenicillin)	Narrow		Both	
	Phenoxymethylpenicillin (Penicillin V)	Narrow		Both	
	Piperacillin	Broad		Both	
	Pivampicillin	Broad		Both	
	Pivmecillinam hydrochloride	Narrow		Both	
	Procaine penicillin	Narrow		Both	
	Ticarcillin	Broad		Both	
**Tetracyclines**					
	Demeclocycline hydrochloride	Broad		Both	
	Doxycycline	Broad		Both	
	Lymecycline	Broad		Both	
	Minocycline hydrochloride	Broad		Both	
	Rolitetracycline	Broad		Both	
	Tetracycline	Broad		Both	
	Tetracycline hydrochloride	Broad		Both	

### Definition of Health Outcomes

The selected health outcomes of the study population are determined through linkage with the following data collections: (1) hospital discharges, (2) pharmaceutical data, (3) nonadmitted patient collection, and (4) laboratory claims, for the period starting from birth or end of antibiotic exposure period of each child to the end of 2021. The specific case definitions, including the International Classification of Diseases, Tenth Revision codes corresponding to the health outcomes under consideration, are provided in Table S1 of Multimedia Appendix 1 [32-35]. To ascertain the prevalence of T1D, we used 3 distinct algorithms to identify cases, facilitating a comprehensive comparison of results to ensure consistency. For IBD, the identification of cases, as outlined in Table S1 of Multimedia Appendix 1, will be subject to further validation against several cohorts of patients with IBD obtained from collaborating gastroenterologists. This validation will involve exploring various combinations of medications prescribed for IBD, including those listed in Table S1 of Multimedia Appendix 1. The analysis aims to provide insights into the diversity of medication regimens associated with IBD cases. Any refinements or enhancements to the algorithms as well as insights gained from the medication combination analysis will be documented and incorporated into the final analysis.

### Other Variables

Fixed covariates/confounders that will be considered in the analyses include sex, ethnicity, deprivation index (based on mesh block) [36], birth weight, gestation, mode of delivery, rurality, and maternal age. Time-dependent covariates include hospitalization for infections and other chronic diseases and selected prescription medications (eg, paracetamol, antivirals, antifungals).

### Follow-Up of Vital Status and New Zealand Residency

Linkage to border movements and mortality data are used to determine whether cohort members are still alive and are based in New Zealand. Those who have emigrated or died prior to their fifth birthday are excluded from the analyses; the follow-up time for those who died or emigrated after the fifth birthday is censored up to that point, which means that the event of interest or health outcome being investigated may not be observed for some individuals.

### Statistical Analysis

To date, the primary focus has been on data preparation that consisted of (1) constructing the cohort through data linkage; (2) identifying the antibiotic exposure variables for all cohort members; (3) identifying other variables, including confounders for all cohort members; and (4) identifying T1D, ADHD, and IBD cases within the cohort using the definitions described in Table S1 of Multimedia Appendix 1. The next stage involves analyses that focus on assessing associations between antibiotic use and the health outcomes described above. For this, we will use Cox proportional hazards regression, with attained age as the analysis time scale. As noted before, children have been followed up until the estimated date of diagnosis, emigration from New Zealand, death, or the end of the study period (December 31, 2021), whichever comes first. 

For each health outcome, analyses will be conducted to measure associations with antibiotic use during specific early life periods (pregnancy, ≤1 year, ≤2 years, and ≤5 years, as well as combinations of these periods). Antibiotic use will be based on the number of prescriptions, which will enable the assessment of dose-response associations. In addition to considering all antibiotic classes combined, we will also conduct analyses where antibiotics will be grouped into different classes/categories (Table 2); this will provide insights into which specific groups of antibiotics may be most strongly associated with the 3 outcomes of interest. Analyses will be stratified by mode of delivery to assess whether associations may be different in different subgroups (effect modification) as has been shown for cesarean section births, with larger effect sizes shown for associations between antibiotic use and T1D for caesarean section births [20,37]. In addition to stratified analyses, we will also assess the role of potential confounders such as sex, prioritized ethnicity, deprivation index, and rurality by using multivariable analyses.

Nested case-control analyses will be conducted as an additional way to address potential bias and confounding. Controls will be matched to cases on year and month of birth, sex, ethnicity, and other potential confounding factors such as residence and deprivation. In addition, to address potential medical surveillance bias, matched controls that occur in the same data collections as the cases will be selected. Nested case-control analyses will also enable the evaluation of possible reverse causation (ie, the health outcome of interest resulting in the prescription of antibiotics rather than the other way around) by disregarding antibiotic use in the 6 months before diagnosis of the cases and the equivalent time point of the matched controls. Moreover, control for confounding by maternal factors will be achieved through within-mother analysis of disease-discordant pairs of siblings. Factors remaining constant between pregnancies could, for example, be the mother’s attitude toward antibiotic prescriptions as well as the general practitioner’s antibiotic prescription practices, which will influence the child’s exposure to antibiotics; other types of analyses can typically not adjust for this. Further, confounding by indication, where the reason for prescribing antibiotics may be linked to the development of chronic conditions, will only be addressed once primary care data become available, enhancing the rigor of the study.

### Study Power

Based on national and international data, we have assumed that at least half of the children will have been prescribed antibiotics within the first year of life (52% in a Finnish study, 15% for specific antibiotic classes [38]). Based on age-specific statistics of the Virtual Diabetes Register, which is an annually updated national register of all patients with diabetes mellitus from 2010 to 2015, we estimate that 400 cases of T1D can be identified within the cohort [39]. Furthermore, based on hospitalization data, as per the age-specific data, we estimate that at least 200 IBD cases (170 cases of Crohn disease and 30 cases of ulcerative colitis) can be included in the study based on hospitalization data. Finally, for ADHD, and as noted earlier, medication dispensing has doubled from 516 per 100,000 in 2007-2008 to 996 per 100,000 in 2016-2017 in New Zealand [9]. Although a breakdown by age group is not available, we estimate that at least 1000 cases can be identified in the cohort. This is a conservative estimate based on our experience with other IDI projects [34], and it is likely that this number is substantially higher (up to 3% of the study population). Thus, we assume that case sets will have a minimum size of 1000 for ADHD, 400 for T1D, and 200 for IBD. Hazard ratio (HR) estimates that are detectable with 80% power (*P*<.05, 2-sided) under different population size and exposure prevalence scenarios are summarized in Table 3. Assuming an exposure prevalence of 33%, this study has 80% power to detect an HR of 1.2 for ADHD, 1.4 for T1D, and 1.5 for IBD. Analyses of specific strata of the study population (eg, based on sex or ethnicity) will have sufficient study power to detect similar effect sizes (Table 3). Considering a lower exposure frequency of 10% (eg, for specific antibiotic classes), the study has 80% power to detect an HR of 1.3 for ADHD, 1.5 for T1D, and 1.7 for IBD. Assuming a further reduction in exposure frequency of 5% (eg, for specific antibiotics), the study has 80% power to detect an HR of 1.5 for ADHD, 1.7 for T1D, and 2.0 for IBD (Table 3).

**Table 3:** Study power: hazard ratio detectable (power 80%, *P*<.05, 2-sided) under different population size and exposure prevalence scenarios.

Strata	Study population (N)	Scenarios for attention-deficit/hyperactivity disorder						Scenarios for type 1 diabetes								Scenarios for inflammatory bowel disease					
		Cases (n)	Exposure prevalence (hazard ratio)					Cases (n)		Exposure prevalence (hazard ratio)					Cases (n)			Exposure prevalence (hazard ratio)			
			50%	33%	10%	5%			50%		33%	10%	5%				50%		33%	10%	5%
All	300,000	1000	1.2	1.2	1.3	1.5	400		1.3		1.4	1.5	1.7	200			1.5		1.5	1.7	2.0
Sex	150,000	500	1.3	1.3	1.5	1.6	200		1.5		1.5	1.7	2.0	100			1.7		1.7	2.0	2.4
Ethnicity	60,000	200	1.5	1.5	1.7	2.0	80		1.8		1.8	2.2	2.5	40			2.2		2.2	2.7	3.2

### Ethics Approval

This study was approved by Human Research Ethics Committee of the University of Otago (reference: HD21/053). Microdata access approval for the project was provided by Statistics New Zealand. To ensure confidentiality, all data are deidentified before access. Access to linked datasets is limited to authorized personnel, and data handling follows ethical guidelines. Sensitive data will be managed securely. Although the data within IDI are fully deidentified, there are several requirements that govern the use of IDI data that this study will adhere to. These are (1) statistical outputs can only be disseminated after outputs have been checked and approved by Statistics New Zealand, (2) the IDI confidentiality rules require the suppression of counts and associated results of analyses on samples smaller than 6, and (3) the random rounding of counts can be up or down to the next multiple of 3.

## Results

As of September 2024, we have completed the data linkage, involving the integration of antibiotic exposure data with outcome variables and all relevant covariates for 315,789 individuals. Preliminary analyses show that both prenatal and early life antibiotic consumption is associated with T1D. Full analyses for all 3 outcomes, that is, analyses adjusted for potential confounders, stratified by ethnicity and sex, and further sensitivity analyses will be completed by the end of 2025.

## Discussion

Antibiotics are widely used in human populations, particularly children. It is therefore important to assess and quantify potential adverse effects of this exposure at the population level so that they can be balanced against the many benefits of these therapies. We hypothesize that antibiotic use will result in a positive and significant hazard risk for the development of chronic childhood conditions. The unique data infrastructure established through Statistics New Zealand’s IDI provides a robust way of assessing such relationships across a large cohort, consisting of the entire New Zealand population who can be followed up for many years. Findings will be of relevance both locally and internationally, particularly in regions with similarly low antimicrobial resistance rates, such as certain European countries [40]. Additionally, in areas with high pediatric antibiotic usage, such as parts of North America and Asia [41], these insights can inform public health strategies aimed at optimizing antibiotic stewardship.

The proposed series of studies, which are large by international standards and based on detailed, complete, and objectively collected antibiotic prescription data, will provide critical new knowledge regarding the role of antibiotics in the development of common chronic childhood conditions. As such, it will provide an important addition to the limited number of studies conducted in humans and has the potential to contribute to the development of feasible avenues for primary prevention, through, for example, targeted changes in antibiotic use in both quantity and type and targeted use of prebiotics or probiotics. Additionally, this study may be expanded to encompass other conditions such as asthma and allergies, which have also been associated with early life antibiotic exposure [42].

The focus of this study is on the effects of antibiotics on the host, but the results are also relevant to the broader issue of antibiotic overuse and its link with increased microbial resistance. New Zealand has comparatively low rates of antimicrobial resistance [43,44]. However, resistance has steadily increased, and experience gained from other countries suggests that this will become more common in New Zealand, raising patient risk and costs for the health system [45]. Antimicrobial resistance is considered one of the biggest man-made public health threats of modern times [43]. Clinicians and public health researchers have advocated that New Zealand needs to urgently institute a range of measures to significantly reduce antimicrobial consumption [46,47]. A systematic review of interventions aimed at reducing antibiotic prescribing for respiratory tract infections across several settings has led to substantial reductions [48], although efforts in New Zealand have not yet resulted in a decrease of antibiotic consumption [45]. This study may provide additional evidence for the need to reduce (unnecessary) antibiotic consumption both in New Zealand and internationally.

## Appendix

Multimedia Appendix 1 Health outcome classification, corresponding International Classification of Diseases Tenth Revision codes, and case definitions.

## Abbreviations

ADHD: attention-deficit/hyperactive disorder
HR: hazard ratio
IBD: inflammatory bowel disease
IDI: integrated data infrastructure
T1D: type 1 diabetes

## Data Availability

The datasets generated and analyzed for this study are not publicly available. Statistics New Zealand’s integrated data infrastructure microdata are available for research purposes, and access is granted based on meeting the conditions set by Stats New Zealand through a secure laboratory environment.
