# Supplementary material for: Antibiotic Use In Utero and Early Life and Risk of Chronic Childhood Conditions in New Zealand: Protocol for a Data Linkage Retrospective Cohort Study
Source: JMIR Res Protoc. 2025 Feb 28;14:e66184. doi: 10.2196/66184 (PMC11909482; doi:10.2196/66184)
Supplement: Multimedia Appendix 1 [file resprot_v14i1e66184_app1.docx]

**Multimedia Appendix 1**

**Table S1.** Health outcome classification, corresponding International Classification of Diseases, Tenth Revision codes, and case definitions.

| Health outcome of interest, International Classification of Diseases, Tenth Revision, code | | International Classification of Diseases, Tenth Revision code description (Australian modification) | Case definitions |
| --- | --- | --- | --- |
| **Type 1 diabetes** | | | |
|  | E100 | Type 1 diabetes mellitus with hyperosmolarity | Three different algorithms are used to identify cases of T1D.   - Algorithm 1: An individual is identified as a case if they meet one of the following criteria: (1) hospital discharge with one or more ICD-10 codes as outlined in this table OR (2) prescription of an insulin medication during the study period. - Algorithm 2: A base dataset for all possible indications of diabetes (ie, type 1 and type 2 diabetes) is extracted first from the cohort dataset. Further processing is based on a previously defined algorithm [32], which identifies an individual as a case if they meet all of the following criteria during the study period: (1) prescription of an insulin medication; AND (2) not dispensed oral hypoglycemics or α-glucosidase inhibitors; AND (3) hospital discharges without a T2D diagnosis and at least one hospital discharge with a T1D diagnosis; AND (4) did not die during the study period with a T2D diagnosis in death records; AND (5) no hospital discharges with cystic fibrosis, pancreatectomy, or neonatal diabetes mellitus before insulin dispensing. - Algorithm 3: A base dataset for all possible indications of diabetes (ie, type 1 and type 2 diabetes) is extracted first from the cohort dataset. Further processing is based on a previously identified algorithm in “Health Tracker” that was developed by the NZ Ministry of Health [33], which identifies an individual as a T1D case if they meet one of the following criteria during the study period: (1) one or more attendances for NNPAC services under T1D purchase unit codes OR (2) discharge/diagnosis events/prescriptions related to T1D in the National Minimum Dataset/Mental Health Information National Collection/Program for Integration of Mental Health Data/Pharmaceutical Collection. Individuals can also be identified as having T1D based on having discharge or other diagnosis events related to T1D and T2D, along with specific prescription patterns such as no oral hypoglycemic/metformin prescription and one or more insulin prescriptions. Age-specific criteria further refine the classification for individuals aged 0-14 years or 15 years and older based on the distribution of T1D and T2D events.   The year of diagnosis for T1D will be based on the date of the first insulin prescription or date of hospital discharge or first NNPAC attendance. |
|  | E101 | Type 1 diabetes mellitus with acidosis |  |
|  | E102 | Type 1 diabetes mellitus with kidney complication |  |
|  | E103 | Type 1 diabetes mellitus with ophthalmic complication |  |
|  | E104 | Type 1 diabetes mellitus with neurological complication |  |
|  | E105 | Type 1 diabetes mellitus with circulatory complication |  |
|  | E106 | Type 1 diabetes mellitus with other specified complication |  |
|  | E107 | Type 1 diabetes mellitus with multiple complications |  |
|  | E108 | Type 1 diabetes mellitus with unspecified complication |  |
|  | E109 | Type 1 diabetes mellitus without complication |  |
| **Attention-deficit/hyperactive disorder** | | | |
|  | F900 | Disturbance of activity and attention | An individual is identified as a case if they meet one of the following criteria: (1) hospital discharge with one or more of the ICD-10 codes outlined in this table OR (2) 2 or more prescriptions of any one of the following medications: methylphenidate (Concerta, Ritalin, Rubifen), dexamphetamine, or atomoxetine (Strattera) during the study period [34]. The ADHD prescriptions listed above are treatments in New Zealand specific to ADHD but may be also prescribed for the rare childhood condition of narcolepsy. The year of diagnosis will be based on the date of the first ADHD medication prescription or hospital discharge. |
|  | F901 | Hyperkinetic conduct disorder |  |
|  | F908 | Other hyperkinetic disorders |  |
|  | F909 | Hyperkinetic disorder, unspecified |  |
| **Inflammatory bowel disease** | | | |
|  | K500 | Crohn disease of small intestine | An individual is identified as a case if they meet one of the following criteria: (1) hospital discharge with one or more of the ICD-10 codes outlined in this table; OR (2) prescription of medications, including but not limited to combinations involving 5-aminosalicylic acid, corticosteroids, and immunomodulators such as infliximab and adalimumab depending on disease subtype and severity [35].  The year of diagnosis will be based on the date of the first IBD medication prescription or hospital discharge. |
|  | K501 | Crohn disease of large intestine |  |
|  | K508 | Other Crohn disease |  |
|  | K509 | Crohn disease, unspecified |  |
|  | K510 | Ulcerative (chronic) pancolitis |  |
|  | K512 | Ulcerative (chronic) proctitis |  |
|  | K513 | Ulcerative (chronic) rectosigmoiditis |  |
|  | K514 | Inflammatory polyps |  |
|  | K515 | Left-sided colitis |  |
|  | K518 | Other ulcerative colitis |  |
|  | K519 | Ulcerative colitis, unspecified |  |
|  | K523 | Indeterminate colitis |  |
|  | K511 | Ulcerative (chronic) ileocolitis |  |
